# Novel Physical Fitness Fuzzy Evaluation Model for Individual Health Promotion

**DOI:** 10.3390/ijerph19095060

**Published:** 2022-04-21

**Authors:** Kuen-Suan Chen, Tzung-Hua Hsieh

**Affiliations:** 1 Department of Industrial Engineering and Management, National Chin-Yi University of Technology, Taichung 411030, Taiwan; kschen@ncut.edu.tw; 2 Department of Business Administration, Chaoyang University of Technology, Taichung 413310, Taiwan; 3 Institute of Innovation and Circular Economy, Asia University, Taichung 413305, Taiwan; 4 Department of Leisure Service Management, Chaoyang University of Technology, Taichung 413310, Taiwan

**Keywords:** upper confidence limits, physical fitness, physical fitness indices, fuzzy evaluation table, fuzzy hypothesis testing

## Abstract

Physical fitness level plays a significant role in health promotion. Cardiorespiratory endurance, muscular endurance, muscle power, and flexibility are the four key indicators of physical fitness level, listed as one of the important fields of preventive medicine. Some studies targeted at students, based on statistical inference, have put forward a set of physical fitness evaluation methods to see whether they have reached the level of healthy physical fitness. Testing and monitoring of individual physical fitness takes up little time and requires a small sample dataset; this paper hence proposed an evaluation and analysis model that suits individual physical fitness by means of a fuzzy evaluation method suitable for evaluating small sample datasets. This paper developed the evaluation model based on the upper confidence limit of the physical fitness evaluation index so that it could reduce the risk of misjudgment caused by sampling error. At the same time, a simple and easy-to-use fuzzy evaluation form was developed as an evaluation interface, which can present the whole picture of all evaluation indicators as well as have good and convenient management performance. Accordingly, it can help every individual simultaneously monitor multiple physical fitness indicators to ensure that each physical fitness index can meet the requirement of healthy physical fitness.

## 1. Introduction

Some studies have pointed out that the boost of physical fitness level has been listed as one of the important fields of preventive medicine and has played a relatively critical role in health promotion [1,2]. With the development and rapid evolution of technologies such as the Internet of Things (IOT) and big data analysis, rapid data analysis technology has gradually matured as well. Under such circumstances, innovation in various industries around the world is pushed forward. The health industry and various manufacturing industries are also moving toward the goal of smart manufacturing by integrating and applying related technologies [3,4,5,6]. Therefore, real-time measurement and monitoring of human health-related information, including blood pressure and heartbeat, have become so popular that the sudden onset of various cardiovascular diseases can be prevented appropriately and in a timely manner, which has a significant impact on the body and mind.

In addition to the above-mentioned health monitoring, taking the initiative to improve the level of physical fitness with moderate physical activity and exercise training is one of the more proactive actions for the pursuit of physical and mental health [2,7]. Plenty of studies related to physical fitness have confirmed that lack of regular exercise is likely to reduce the basal metabolic rate and cause obesity. It will also increase the risk of cardiovascular disease and chronic diseases [8,9,10]. Meanwhile, it will also increase cardiovascular diseases so that body function will decline early and then various chronic diseases will occur; furthermore, it will seriously affect the health of the world [11,12,13]. In contrast, moderate exercise can not only increase physical fitness but can also benefit the overall health maintenance and life quality improvement of individuals [14,15,16,17]. Obviously, evaluating physical fitness is important for individuals, and it plays an extremely important role in health promotion, being one of the significant fields of preventive medicine [18,19,20,21,22].

In addition to individual physical fitness, participating in moderate exercise and recreational sport can be beneficial to our health condition. In the field of medical science, blood tests and urinalysis are commonly used to diagnose a person’s health condition [1]. In contrast to the test and diagnostic model used in medical science, this paper adopts the five physical fitness indicators (PFI) proposed by Lin et al. [2,7]: cardiorespiratory endurance, muscular endurance, muscular power, flexibility, and body mass index. These five individual physical fitness indicators are mainly used to measure the state of all students’ physical fitness and serve as a policy reference for the government to improve students’ physical fitness. Nevertheless, with the arrival of the Industry 4.0 era, human beings enjoy a more convenient and comfortable work life, but also an increase in workload and work pressure [1]. Meanwhile, occupational activity has also changed into the sedentary lifestyle of a high-tech civilization. As a result, many people have begun to focus on leisure time activities and regular exercise to enhance their individual physical fitness [7]. As the testing and monitoring of individual physical fitness requires only a small amount of time and has a small sample dataset, this paper proposes a physical fitness evaluation and analysis model fitting individuals through a fuzzy evaluation method suitable for evaluating a small sample dataset. As the body mass index will not change significantly in a short period of time [23,24], the physical fitness evaluation and analysis model developed in this paper includes (1) cardiorespiratory endurance, (2) muscular endurance, (3) muscle power, and (4) flexibility. These four physical fitness indicators are described below [2,7].

(1) Cardiorespiratory endurance index: Cardiorespiratory endurance belongs to the smaller-the-better quality characteristic [2,7]. Let random variable $X_{1}$ represent the time that the subject has spent completing the specified running distance and $U_{1}$ represent the upper time limit of completing the test. It is assumed that $X_{1}$ is distributed as the normal distribution with mean $\mu_{1}$ and standard deviation $\sigma_{1}$, denoted as $X_{1}\sim N\left(\mu_{1},\sigma_{1}^{2}\right)$. Then, the cardiorespiratory endurance index is expressed as follows:

(1) $$P_{F1}=\frac{U_{1}-\mu_{1}}{\sigma_{1}}$$

(2) Muscular endurance index: Muscular endurance belongs to the larger-the-better quality characteristic [2,7]. Let random variable $X_{2}$ represent the number of times the subject completes sit-ups with knees bent within the specified time and $L_{2}$ represent the lower limit of the number of times the subject completes sit-ups with knees bent within the specified time. It is assumed that $X_{2}$ is distributed as $N\left(\mu_{2},\sigma_{2}^{2}\right)$. Then, the muscular endurance index $P_{F2}$ is expressed as follows:

(2) $$P_{F2}=\frac{\mu_{2}-L_{2}}{\sigma_{2}}$$

(3) Muscular power index: Muscular power belongs to the larger-the-better quality characteristic [2,7]. Let random variable $X_{3}$ represent the distance that the subject can jump when completing the standing long jump and $L_{3}$ represent the lower limit of the distance that the subject can jump when completing the standing long jump. It is assumed that $X_{3}$ is distributed as $N\left(\mu_{3},\sigma_{3}^{2}\right)$. Then, the muscular endurance index is defined as follows:

(3) $$P_{F3}=\frac{\mu_{3}-L_{3}}{\sigma_{3}}$$

(4) Flexibility index: Flexibility belongs to the larger-the-better quality characteristic [2,7]. Let random variable $X_{4}$ represent the stretch distance of seated forward flexion that the subject completes and $L_{4}$ represent the lower limit of the stretch distance of seated forward flexion that the subject completes. It is assumed that $X_{4}$ is distributed as $N\left(\mu_{4},\sigma_{4}^{2}\right)$. Then, the muscular endurance index is expressed as follows:

(4) $$P_{F4}=\frac{\mu_{4}-L_{4}}{\sigma_{4}}$$

According to Lin et al. [2], the ratio $\left(r_{i}\right)$ that these four physical fitness indices $\left(P_{Fi}\right)$ all meet the basic requirements of physical fitness has a one-to-one mathematical relationship as follows:

(5) $$r_{i}=\Phi \left(P_{Fi}\right)$$

Next, this paper applies the fuzzy testing method based on the upper confidence limit to evaluate whether these four physical fitness indicators of the subject meet the basic health requirements. The purpose of this paper is for people to perform the four fitness tests—cardiorespiratory endurance, muscular endurance, muscle power, and flexibility—on their own; using the method developed in this paper, people can exam their physical conditions, make adjustment to their way of living, and achieve their health goals. The model developed in this paper can apply to more than the evaluation of physical fitness. In addition, all kinds of previously mentioned clinical test data can be used to develop a similar evaluation model, providing medical professions with reference information for making diagnoses.

The other sections of this paper are organized as follows. In Section 2, we derive the 100 (1 − α)% upper confidence limits for these four physical fitness indices. In Section 3, this study proposes a confidence-interval-based fuzzy testing method to evaluate the physical fitness and determine whether the physical fitness needs to improve. An application example is presented in Section 4 to demonstrate the applicability of the proposed approach. Finally, Section 5 provides the conclusions.

## 2. Upper Confidence Limits for Physical Fitness Indices

Let $\left(X_{h,1},\cdots ,X_{h,j},\cdots ,X_{h,n}\right)$ be the test sample data of a subject’s four physical fitness indices, where *h* = 1, 2, 3, 4. Then, the sample mean and sample standard deviation can be shown respectively as follows:

(6) $${\bar{X}}_{h}=\frac{1}{n}\times \sum_{j=1}^{n}X_{h,j}$$

and

(7) $$S_{h}=\sqrt{\frac{1}{n}\times \sum_{j=1}^{n}{\left(X_{h,j}-{\bar{X}}_{h}\right)}^{2}}$$

Then, the estimators of the four physical fitness indices can be displayed as follows:

(8) $$P_{Fh}^{*}=\left\{\begin{array}{l} \frac{U_{h}-{\bar{X}}_{h}}{S_{h}},\;h=1\\ \frac{{\bar{X}}_{h}-L_{h}}{S_{h}},\;h=2,3,4 \end{array}\right.$$

Under the assumption of normality, let

(9) $$Z_{h}=\left\{\begin{array}{l} \frac{\sqrt{n}\left[\left(U_{h}-\mu_{h}\right)-\left(U_{h}-{\bar{X}}_{h}\right)\right]}{\sigma_{h}},\;h=1\\ \frac{\sqrt{n}\left[\left({\bar{X}}_{h}-L_{h}\right)-\left(\mu_{h}-L_{h}\right)\right]}{\sigma_{h}},\;h=2,3,4 \end{array}\right.$$

and

(10) $$K_{h}=\frac{nS_{h}^{2}}{\sigma_{h}^{2}}$$

then, *Z_h_* is distributed as a standard normal distribution with mean $\mu_{h}$ and standard deviation $\sigma_{h}/\sqrt{n}$, and $K_{h}$ is distributed as a chi-square distribution with n−1 degree of freedom, denoted as $\chi_{n-1}^{2}$. To derive the 1−α upper confidence limit on index $P_{F1}$, we have $p\left\{K_{1}\leq \chi_{1-\alpha /2;n-1}^{2}\right\}=1-\alpha /2$ and $p\left\{Z_{h}\leq Z_{\alpha /2}\right\}=1-\alpha /2$, where $\chi_{1-\alpha /2;n-1}^{2}$ is the lower 1−α/2 quantile of $\chi_{n-1}^{2}$ and $Z_{\alpha /2}$ is the upper α/2 quantile of $N\left(0,1\right)$. Then,

(11) $$p\left\{\frac{1}{\sigma_{1}}\leq \frac{1}{S_{1}}\sqrt{\frac{\chi_{1-\alpha /2;n-1}^{2}}{n}}\right\}=1-\frac{\alpha }{2}$$

and

(12) $$p\left\{P_{F1}\leq P_{F1}^{*}\times \left(\frac{S_{1}}{\sigma_{1}}\right)+\frac{Z_{\alpha /2}}{\sqrt{n}}\right\}=1-\frac{\alpha }{2}$$

Similarly, to derive the 1−α upper confidence limits on index $P_{Fh}$, we have $p\left\{K_{h}\leq \chi_{1-\alpha /2;n-1}^{2}\right\}=1-\alpha /2$ and $p\left\{Z_{h}\geq -Z_{\alpha /2}\right\}=1-\alpha /2$, h=2,3,4. Then,

(13) $$p\left\{\frac{1}{\sigma_{h}}\leq \frac{1}{S_{h}}\sqrt{\frac{\chi_{1-\alpha /2;n-1}^{2}}{n}}\right\}=1-\frac{\alpha }{2}$$

and

(14) $$p\left\{P_{Fh}\leq P_{Fh}^{*}\times \left(\frac{S_{h}}{\sigma_{h}}\right)+\frac{Z_{\alpha /2}}{\sqrt{n}}\right\}=1-\frac{\alpha }{2}$$

Additionally, let event $A_{h}$ and event $B_{h}$ be displayed as follows:

(15) $$A_{h}=\left\{\frac{1}{\sigma_{h}}\leq \frac{1}{S_{h}}\sqrt{\frac{\chi_{1-\alpha /2;n-1}^{2}}{n}}\right\}$$

and

(16) $$B_{h}=\left\{P_{Fh}\leq P_{Fh}^{*}\times \left(\frac{S_{h}}{\sigma_{h}}\right)+\frac{Z_{\alpha /2}}{\sqrt{n}}\right\}$$

Then, the compliments of event $A_{h}$ and event $B_{h}$ can be shown as follows:

(17) $$A_{h}^{c}=\left\{\frac{1}{\sigma_{h}}>\frac{1}{S_{h}}\sqrt{\frac{\chi_{1-\alpha /2;n-1}^{2}}{n}}\right\}$$

and

(18) $$B_{h}^{c}=\left\{P_{Fh}>P_{Fh}^{*}\times \left(\frac{S_{h}}{\sigma_{h}}\right)+\frac{Z_{\alpha /2}}{\sqrt{n}}\right\}$$

Based on DeMorgan’s rule and Boole’s inequality, then we have

(19) $$p\left(A_{h}\cap B_{h}\right)\geq 1-p\left(A_{h}^{c}\right)-p\left(A_{h}^{c}\right)=1-\alpha$$

Therefore,

(20) $$p\left\{P_{Fh}\leq P_{Fh}^{*}\times \left(\frac{S_{h}}{\sigma_{h}}\right)+\frac{Z_{\alpha /2}}{\sqrt{n}},\frac{1}{\sigma_{h}}\leq \frac{1}{S_{h}}\sqrt{\frac{\chi_{1-\alpha /2;n-1}^{2}}{n}}\right\}\geq 1-\alpha \;\Rightarrow p\left\{P_{Fh}\leq P_{Fh}^{*}\times \sqrt{\frac{\chi_{1-\alpha /2;n-1}^{2}}{n}}+\frac{Z_{\alpha /2}}{\sqrt{n}}\right\}\geq 1-\alpha$$

The 1−α upper confidence limits on index $P_{Fh}$ is

(21) $$UP_{Fh}=P_{Fh}^{*}\times \sqrt{\frac{\chi_{1-\alpha /2;n-1}^{2}}{n}}+\frac{Z_{\alpha /2}}{\sqrt{n}}$$

Let ${\bar{x}}_{h}$ and $s_{h}$ be the observed values of ${\bar{X}}_{h}$ and $S_{h}$, respectively, as follows:

(22) $${\bar{x}}_{h}=\frac{1}{n}\times \sum_{j=1}^{n}x_{h,j}$$

and

(23) $$s_{h}=\sqrt{\frac{1}{n}\times \sum_{j=1}^{n}{\left(x_{h,j}-{\bar{x}}_{h}\right)}^{2}}$$

Thus, the observed value of $P_{Fh}^{*}$ and the upper confidence limit $UP_{Fh}$, respectively, are expressed as follows:

(24) $$P_{Fh0}^{*}=\left\{\begin{array}{l} \frac{U_{h}-{\bar{x}}_{h}}{s_{h}},\;h=1\\ \frac{{\bar{x}}_{h}-L_{h}}{s_{h}},\;h=2,3,4 \end{array}\right.$$

and

(25) $$UP_{Fh0}=P_{Fh0}^{*}\times \sqrt{\frac{\chi_{1-\alpha /2;n-1}^{2}}{n}}+\frac{Z_{\alpha /2}}{\sqrt{n}}$$

## 3. Fuzzy Hypothesis Testing

The fuzzy evaluation is an effective approach to evaluate whether the physical fitness of the subject is acceptable [25,26]. As noted by Lin et al. [2], the hypothesis for testing at a significant level can be stated as follows:

Null hypothesis $H_{0}:P_{Fh}\geq k$ (the performance of physical fitness is acceptable);

Alternative hypothesis $H_{1}:P_{Fh}<k$ (the performance of physical fitness is unacceptable).

As described by Chen [27], the α-cuts of the triangular-shaped fuzzy number ${\tilde{P}}_{Fh}$ is expressed as follows:

(26) $${\tilde{P}}_{Fh}\left[\alpha \right]=\left\{\begin{array}{l} \left[P_{Fh}\left(1\right),P_{Fh}\left(\alpha \right)\right],\;\mathrm{for}\;0.05\leq \alpha \leq 1\\ \left[P_{Fh}\left(1\right),P_{Fh}\left(0.05\right)\right],\;\mathrm{for}\;0\leq \alpha \leq 0.05 \end{array}\right.$$

where

(27) $$P_{Fh}\left(1\right)=P_{Fh0}^{*}\times \sqrt{\frac{\chi_{0.5;n-1}^{2}}{n}}$$

(28) $$P_{Fh}\left(\alpha \right)=P_{Fh0}^{*}\times \sqrt{\frac{\chi_{1-\alpha /2;n-1}^{2}}{n}}+\frac{Z_{\alpha /2}}{\sqrt{n}}$$

Therefore, the half-triangular-shaped fuzzy number is ${\tilde{P}}_{Fh}$$=\Delta \left(P_{FhM},P_{FhR}\right)$, where

(29) $$P_{FhM}=P_{Fh0}^{*}\times \sqrt{\frac{\chi_{0.5;n-1}^{2}}{n}}$$

(30) $$P_{FhR}=P_{Fh0}^{*}\times \sqrt{\frac{\chi_{0.975;n-1}^{2}}{n}}+\frac{Z_{0.025}}{\sqrt{n}}$$

Thus, the membership function of ${\tilde{P}}_{Fh}$ is

(31) $$\eta_{h}\left(x\right)=\left\{\begin{array}{l} 0\;if\;x<P_{FhM}\\ 1\;if\;x=P_{FhM}\\ \alpha \;if\;P_{FhM}<x<P_{FhR}\\ 0\;if\;x\geq P_{FhR} \end{array}\right.$$

where α is determined by

(32) $$x=P_{Fh0}^{*}\times \sqrt{\frac{\chi_{1-\alpha /2;n-1}^{2}}{n}}+\frac{Z_{\alpha /2}}{\sqrt{n}}$$

**Figure 1:**
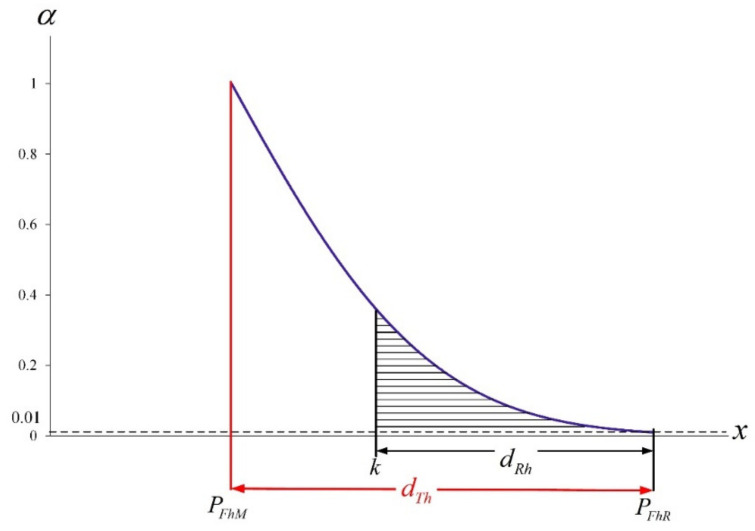
Membership function $\eta_{h}\left(x\right)$ with vertical line *x* =  *k*.

The diagram of membership function $\eta_{h}\left(x\right)$ with vertical line *x* = *k* is presented in Figure 1 as follows:

Let set $A_{Th}$ be the area in the graph of $\eta_{h}\left(x\right)$, then

(33) $$A_{Th}=\left\{\left.\left(x,\alpha \right)\right|P_{FhM}\left(\alpha \right)\leq x\leq P_{FhR}\left(\alpha \right),0\leq \alpha \leq 1\right\}$$

Similarly, let set $A_{Rh}$ be the area in the graph of $\eta_{h}\left(x\right)$ but to the right of the vertical line *x* = *k*, then

(34) $$A_{Rh}=\left\{\left.\left(x,\alpha \right)\right|k\leq x\leq P_{FhR}\left(\alpha \right),0\leq \alpha \leq 1\right\}$$

Based on Chen et al. [28] and Yu et al. [29], this paper simplified Buckley’s method [30] to replace $A_{Rh}/A_{Th}$ with $d_{Rh}/d_{Th}$ to perform fuzzy testing. Moreover, $d_{Rh}=P_{FhR}-k$ and $d_{Th}=P_{FhR}-P_{FhM}$ are expressed as follows:

(35) $$d_{Rh}=P_{FhR}-k=P_{Fh0}^{*}\times \sqrt{\frac{\chi_{0.995;n-1}^{2}}{n}}+\frac{Z_{0.005}}{\sqrt{n}}-k$$

(36) $$d_{Th}=P_{FhR}-P_{FhM}=P_{Fh0}^{*}\times \sqrt{\frac{\chi_{0.995;n-1}^{2}}{n}}+\frac{Z_{0.005}}{\sqrt{n}}-P_{Fh0}^{*}\times \sqrt{\frac{\chi_{0.5;n-1}^{2}}{n}}$$

Based on Equations (35) and (36), $d_{Rh}/d_{Th}$ can be shown as follows:

(37) $$d_{Rh}/d_{Th}=\frac{P_{FhR}-k}{P_{FhR}-P_{FhM}}=\frac{P_{Fh0}^{*}\times \sqrt{\frac{\chi_{0.995;n-1}^{2}}{n}}+\frac{Z_{0.005}}{\sqrt{n}}-k}{P_{Fh0}^{*}\times \left(\sqrt{\frac{\chi_{0.995;n-1}^{2}}{n}}+\frac{Z_{0.005}}{\sqrt{n}}-\sqrt{\frac{\chi_{0.5;n-1}^{2}}{n}}\right)}$$

Therefore, we let $0\;<\;\phi_{1}<\phi_{2}<1.0$. As noted by Yu et al. [31], we may obtain the following fuzzy testing rules:

(1) If $d_{Rh}/d_{Th}$≤$\phi_{1}$, then reject $H_{0}$ and conclude $P_{Fh}<k$, indicating that the individual physical fitness must be leveled up to meet the requirement of healthy physical fitness.
(2) If $\phi_{1}$<$d_{Rh}/d_{Th}$<$\phi_{2}$, then make no decision on whether to reject/not reject $H_{0}$, showing that it needs to be re-evaluated.
(3) If $\phi_{2}$≤$d_{Rh}/d_{Th}$<1.0, then do not reject $H_{0}$ and conclude $P_{Fh}\geq k$, demonstrating that the individual physical fitness has reached the requirement and can keep unchanged.

In order to make the fuzzy test more convenient, this paper summarized the testing statistics and index estimates of four physical fitness items as shown in Table 1.

Next, based on the relevant information in Table 1 and Equations (29), (30) and (35)–(37), this study established a fuzzy evaluation table for physical fitness as displayed in Table 2.

Then, according to the value of $d_{Rh}/d_{Th}$ calculated in Table 2, the decision can be made based on the above fuzzy testing rules, and the relevant action plan can be proposed as well.

## 4. An Illustrative Example

In order to illustrate the practical application of the fuzzy evaluation method of physical fitness proposed in Section 3, this paper assumed that an 18-year-old male student was under the pressure of further education in Taiwan and wanted to know about his physical fitness status; except for regular activities and moderate exercise, four items of physical fitness were tested from Monday to Friday for 2 weeks, 10 days in total. According to the study of Lin et al. [2] and the physical fitness norm of Taiwan, the testing methods and standards for the four physical fitness items of the 18-year-old male student are described as follows:

(1) Cardiorespiratory endurance: The testing method and standard of cardiorespiratory endurance refer to the seconds in which the subject can run and walk 1600 m. According to the physical fitness norm in Taiwan, the basic requirement for the intermediate level of this test for an 18-year-old male student is to complete the task in 598 s ($L_{1}=$ 598). Test data and related statistics for 10 tests are listed below:

589, 593, 590, 591, 589, 590, 589, 589, 592, 594;

$${\bar{x}}_{1}=\frac{1}{10}\times \sum_{j=1}^{10}x_{1,j}=590.6\;s_{1}=\sqrt{\frac{1}{10}\times \sum_{j=1}^{10}{\left(x_{1,j}-{\bar{x}}_{1}\right)}^{2}}=1.838\;P_{F10}^{*}=\frac{U_{1}-{\bar{x}}_{1}}{s_{1}}=4.03$$

(2) Muscular endurance: The testing method and standard of muscular endurance refer to the number of times the subject completes sit-ups with knees bent within one minute. According to the physical fitness norm of Taiwan, the basic requirement of the average level of this test for an 18-year-old male student is to complete the task at least 33 times in a minute ($L_{2}=$ 33). The test data and related statistics for 10 tests are listed as follows:

39, 38, 37, 38, 37, 39, 38, 37, 37, 38;

$${\bar{x}}_{2}=\frac{1}{10}\times \sum_{j=1}^{10}x_{2,j}=37.8\;s_{2}=\sqrt{\frac{1}{10}\times \sum_{j=1}^{10}{\left(x_{2,j}-{\bar{x}}_{2}\right)}^{2}}=0.789\;P_{F20}^{*}=\frac{{\bar{x}}_{2}-L_{2}}{s_{2}}=6.09$$

(3) Muscular power: The testing method and standard of muscular power refer to the distance of standing long jump that the subject can complete. According to Taiwan’s physical fitness norm, the basic requirement for the intermediate level of this test for an 18-year-old male student is that the standing long jump distance is at least 185 cm ($L_{3}=$ 185). The test data and related statistics for 10 tests are seen as follows:

191, 190, 191, 190, 191, 189, 188, 192, 191, 190;

$${\bar{x}}_{3}=\frac{1}{10}\times \sum_{j=1}^{10}x_{3,j}=192\;s_{3}=\sqrt{\frac{1}{10}\times \sum_{j=1}^{10}{\left(x_{3,j}-{\bar{x}}_{3}\right)}^{2}}=1.160\;P_{F30}^{*}=\frac{{\bar{x}}_{3}-L_{3}}{s_{3}}=6.04$$

(4) Flexibility: The testing method and standard of flexibility refer to the stretch distance of seated forward flexion that the subject can complete. According to Taiwan’s physical fitness norm, the basic requirement for the average level of this test for an 18-year-old male student is that the stretch distance is at least 18 cm ($L_{4}=$ 18). Test data and related statistics for 10 tests are seen as follows:

18.5, 18.6, 18.4, 18.7, 18.6, 18.5, 18.6, 18.7, 18.6, 18.9;

$${\bar{x}}_{4}=\frac{1}{10}\times \sum_{j=1}^{10}x_{4,j}=18.59\;s_{4}=\sqrt{\frac{1}{10}\times \sum_{j=1}^{10}{\left(x_{4,j}-{\bar{x}}_{4}\right)}^{2}}=0.099\;P_{F40}^{*}=\frac{{\bar{x}}_{4}-L_{4}}{s_{4}}=5.93$$

Subsequently, according to Table 1 in Section 3, the mean, standard deviation, and index estimates of physical fitness (n= 10) of the four physical fitness tests are filled in as shown in Table 3 below:

Based on Lin et al. [2], the hypothesis of testing can be stated as follows:

Null hypothesis $H_{0}:P_{Fh}\geq 6$ (the performance of physical fitness is acceptable);

Alternative hypothesis $H_{1}:P_{Fh}<6$ (the performance of physical fitness is unacceptable).

Then, based on Equations (29), (30) and (35)–(37) and the data in Table 3, we have

Item 1:

$$P_{F1M}=P_{F10}^{*}\times \sqrt{\frac{\chi_{0.5;9}^{2}}{10}}=3.681\;P_{F1R}=P_{F10}^{*}\times \sqrt{\frac{\chi_{0.975;9}^{2}}{10}}+\frac{Z_{0.025}}{\sqrt{10}}=6.178\;d_{R1}=P_{F1R}-6=\;6.178-6=\;0.178\;d_{T1}=P_{F1R}-P_{F1M}=\;6.178-3.681=2.497\;d_{R1}/d_{T1}=\frac{P_{F1R}-6}{P_{F1R}-P_{F1M}}=\frac{0.178}{2.497}=0.071$$

Item 2:

$$P_{F2M}=P_{F20}^{*}\times \sqrt{\frac{\chi_{0.5;9}^{2}}{10}}=5.563\;P_{F2R}=P_{F20}^{*}\times \sqrt{\frac{\chi_{0.975;9}^{2}}{10}}+\frac{Z_{0.025}}{\sqrt{10}}=9.019\;d_{R2}=P_{F2R}-6=\;9.019-6=\;3.019\;d_{T2}=P_{F2R}-P_{F2M}=\;9.019-5.563=3.456\;d_{R2}/d_{T2}=\frac{P_{F2R}-6}{P_{F2R}-P_{F2M}}=\frac{3.019}{3.456}=0.874$$

Item 3:

$$P_{F3M}=P_{F30}^{*}\times \sqrt{\frac{\chi_{0.5;9}^{2}}{10}}=5.517\;P_{F3R}=P_{F30}^{*}\times \sqrt{\frac{\chi_{0.975;9}^{2}}{10}}+\frac{Z_{0.025}}{\sqrt{10}}=8.950\;d_{R3}=P_{F3R}-6=\;8.950-6=\;2.950\;d_{T3}=P_{F3R}-P_{F3M}=\;8.950-5.517=3.433\;d_{R3}/d_{T3}=\frac{P_{F3R}-6}{P_{F3R}-P_{F3M}}=\frac{2.950}{3.433}=0.859$$

Item 4:

$$P_{F4M}=P_{F40}^{*}\times \sqrt{\frac{\chi_{0.5;9}^{2}}{10}}=5.416\;P_{F4R}=P_{F40}^{*}\times \sqrt{\frac{\chi_{0.975;9}^{2}}{10}}+\frac{Z_{0.025}}{\sqrt{10}}=8.799\;d_{R4}=P_{F4R}-6=\;8.799-6=\;2.799\;d_{T4}=P_{F4R}-P_{F4M}=\;8.799-5.416=3.383\;d_{R4}/d_{T4}=\frac{P_{F4R}-6}{P_{F4R}-P_{F4M}}=\frac{2.799}{3.383}=0.827$$

The above information is shown in Table 4 below.

According to Table 4 above, since $d_{Rh}/d_{T1}$ = 0.071 ≤ 0.4, the cardiorespiratory endurance of the male student does not meet the basic requirement of the intermediate level so that his cardiorespiratory endurance must be lifted to meet the requirement of healthy physical fitness. Given that the 95% upper confidence limit of the cardiorespiratory endurance index is 6.178 ($P_{F1R}$ = 6.178), greater than 6, on the basis on the statistical testing rules, $H_{0}$ is not rejected, indicating that the male student’s cardiorespiratory endurance meets the basic requirement of the intermediate level. However, the point estimate of the cardiorespiratory endurance index is $P_{F10}^{*}$ 4.03, far less than the requirement of $H_{0}:P_{Fh}\geq 6$. Obviously, the fuzzy testing evaluation model proposed by this paper is more reasonable than the statistical test, which is consistent with the conclusions of many studies [32,33,34,35].

## 5. Conclusions

Physical fitness is an important index for evaluating the physical fitness of individuals, plays an extremely critical role in health promotion, and is one of the significant fields of preventive medicine. This paper proposed an evaluation and analysis model fitting physical fitness items of individuals via a fuzzy evaluation method suitable for evaluating a small sample dataset. This paper has excluded the body mass index, which does not change significantly in a short period of time. The advantages of this fuzzy evaluation model are presented as follows:

(1) Based on the upper confidence limit, the risk of misjudgment resulting from sampling error can be lowered.
(2) Through the fuzzy testing method based on the upper confidence limit, experts’ past experience can be incorporated into the small samples, and the accuracy of the evaluation can be maintained as well.
(3) The developed fuzzy evaluation table for physical fitness is simple and easy to use.
(4) Using the easy-to-use evaluation table as the evaluation interface, the whole picture of all evaluation indicators is presented, which has good and convenient management performance.

In addition, various blood tests or urinalysis need to be ordered by a clinical doctor, with the test data obtained through professional instruments. However, unlike professional testing and diagnostic models, fitness testing data only need to be measured by individuals in an appropriate place [1]. Therefore, the fuzzy evaluation model proposed in this paper can help every individual monitor four physical fitness indicators at the same time to ensure that each physical fitness index can meet the requirement of healthy physical fitness.

Due to a lack of research on the actual relationship between physical fitness and healthy longevity, it is one of the important issues requiring future research. BMI, in addition, is one of the important indicators in physical fitness tests. However, in such a short period of time, the value will not change significantly [23,24], so the BMI is not included in the model. For longitudinal research, it will then be necessary to include BMI. When the test data show a non-normal distribution, how to revise and improve the model is another important issue in the future. Furthermore, developing a specialized evaluation model targeting all clinical tests using the method proposed in this paper to help medical professionals make more accurate diagnoses will be another critical research issue in the future.

## Figures and Tables

**Table 1:** Testing statistics and index estimates of four physical fitness items.

Items	Physical Fitness	Specifications	${\bar{x}}_{h}$	$s_{h}$	$P_{Fh0}^{*}$
1	Cardiorespiratory endurance	$U_{1}$	${\bar{x}}_{1}$	$s_{1}$	$P_{F10}^{*}$
2	Muscular endurance	$L_{2}$	${\bar{x}}_{2}$	$s_{2}$	$P_{F20}^{*}$
3	Muscular power	$L_{3}$	${\bar{x}}_{3}$	$s_{3}$	$P_{F30}^{*}$
4	Flexibility	$L_{4}$	${\bar{x}}_{4}$	$s_{4}$	$P_{F40}^{*}$

**Table 2:** Fuzzy evaluation table for physical fitness.

Items	$P_{FhM}$	$P_{FhR}$	$d_{Rh}$	$d_{Th}$	${d_{Rh}/d_{Th}}^{***}$
1	$P_{F1M}$	$P_{F1R}$	$d_{R1}$	$d_{T1}$	$d_{Rh}/d_{T1}$
2	$P_{F2M}$	$P_{F2R}$	$d_{R2}$	$d_{T2}$	$d_{Rh}/d_{T2}$
3	$P_{F3M}$	$P_{F3R}$	$d_{R3}$	$d_{T3}$	$d_{Rh}/d_{T3}$
4	$P_{F4M}$	$P_{F4R}$	$d_{R4}$	$d_{T4}$	$d_{Rh}/d_{T4}$

*Remark*: When $d_{Rh}/d_{Th}$≤$\phi_{1}$, mark “***” in the upper right corner, indicating that the individual physical fitness item does not meet the requirement of healthy physical fitness and they must continue to improve their physical fitness.

**Table 3:** Testing statistics and index estimates of the four physical fitness items in the case study.

Items	Physical Fitness	Specifications	${\bar{x}}_{h}$	$s_{h}$	$P_{Fh0}^{*}$
1	Cardiorespiratory endurance	$U_{1}=$ 598	${\bar{x}}_{1}$ = 590.6	$s_{1}$ = 1.838	$P_{F10}^{*}$ = 4.03
2	Muscular endurance	$L_{2}=$ 33	${\bar{x}}_{2}$ = 37.8	$s_{2}$ = 0.789	$P_{F20}^{*}$ = 6.09
3	Muscular power	$L_{3}=$ 185	${\bar{x}}_{3}$ = 192	$s_{3}$ = 1.160	$P_{F30}^{*}$ = 6.04
4	Flexibility	$L_{4}=$ 18	${\bar{x}}_{4}$ = 18.59	$s_{4}$ = 0.099	$P_{F40}^{*}$ = 5.93

**Table 4:** Fuzzy evaluation table for physical fitness.

Items	$P_{FhM}$	$P_{FhR}$	$d_{Rh}$	$d_{Rh}$	${d_{Rh}/d_{Th}}^{***}$
1	$P_{F1M}$= 3.681	$P_{F1R}$= 6.178	$d_{R1}$= 0.178	$d_{T1}$= 2.497	$d_{Rh}/d_{T1}$= 0.071^***^
2	$P_{F2M}$= 5.563	$P_{F2R}$= 9.019	$d_{R2}$= 3.019	$d_{T2}$= 3.456	$d_{Rh}/d_{T2}$= 0.874
3	$P_{F3M}$= 5.517	$P_{F3R}$= 8.950	$d_{R3}$= 2.950	$d_{T3}$= 3.433	$d_{Rh}/d_{T3}$= 0.859
4	$P_{F4M}$= 5.416	$P_{F4R}$= 8.799	$d_{R4}$= 2.799	$d_{T4}$= 3.383	$d_{Rh}/d_{T4}$= 0.827

*Remark*: When $d_{Rh}/d_{Th}$≤$\phi_{1}$= 0.4, “***” is marked in the upper right corner, indicating that the individual physical fitness item does not meet the requirement of healthy physical fitness, and the physical fitness of the item must continue to improve.

## Data Availability

Not applicable.
